# Supplementary material for: Human cortex organizes dynamic co-fluctuations along sensation-association axis
Source: bioRxiv. 2025 Jul 16:2025.07.14.660681. Preprint. [Version 1] doi: 10.1101/2025.07.14.660681 (PMC12338729; doi:10.1101/2025.07.14.660681)
Supplement: Supplement 1 [file NIHPP2025.07.14.660681v1-supplement-1.pdf]

bioRxiv preprint doi: https://doi.org/10.1101/2025.07.14.660681; this version posted July 16, 2025. The copyright holder for this preprint (which was not certified by peer review) is the author/funder, who has granted bioRxiv a license to display the preprint in perpetuity. It is made available under aCC-BY 4.0 International license.

Supplementary Materials

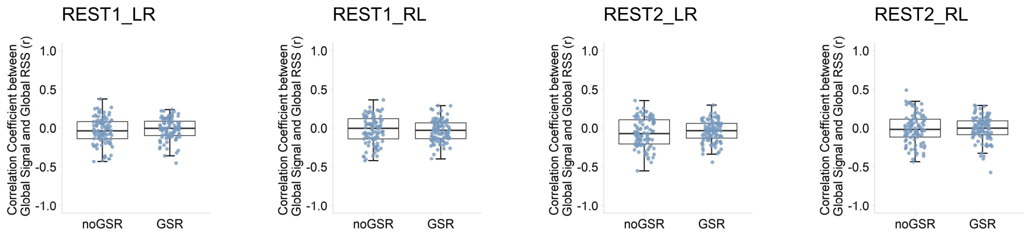

Fig. S1. The relationship between global signal and global RSS. We calculated Pearson's correlation coefficient between global signal and global RSS for four scan sessions of each subject. 'noGSR' refers to the condition that global RSS was calculated without global signal regression, whereas 'GSR' indicates that global RSS was calculated after global signal regression. There was no significant difference between the conditions of noGSR and GSR, which suggests global signal is not directly related to global RSS.

bioRxiv preprint doi: https://doi.org/10.1101/2025.07.14.660681; this version posted July 16, 2025. The copyright holder for this preprint (which was not certified by peer review) is the author/funder, who has granted bioRxiv a license to display the preprint in perpetuity. It is made available under aCC-BY 4.0 International license.

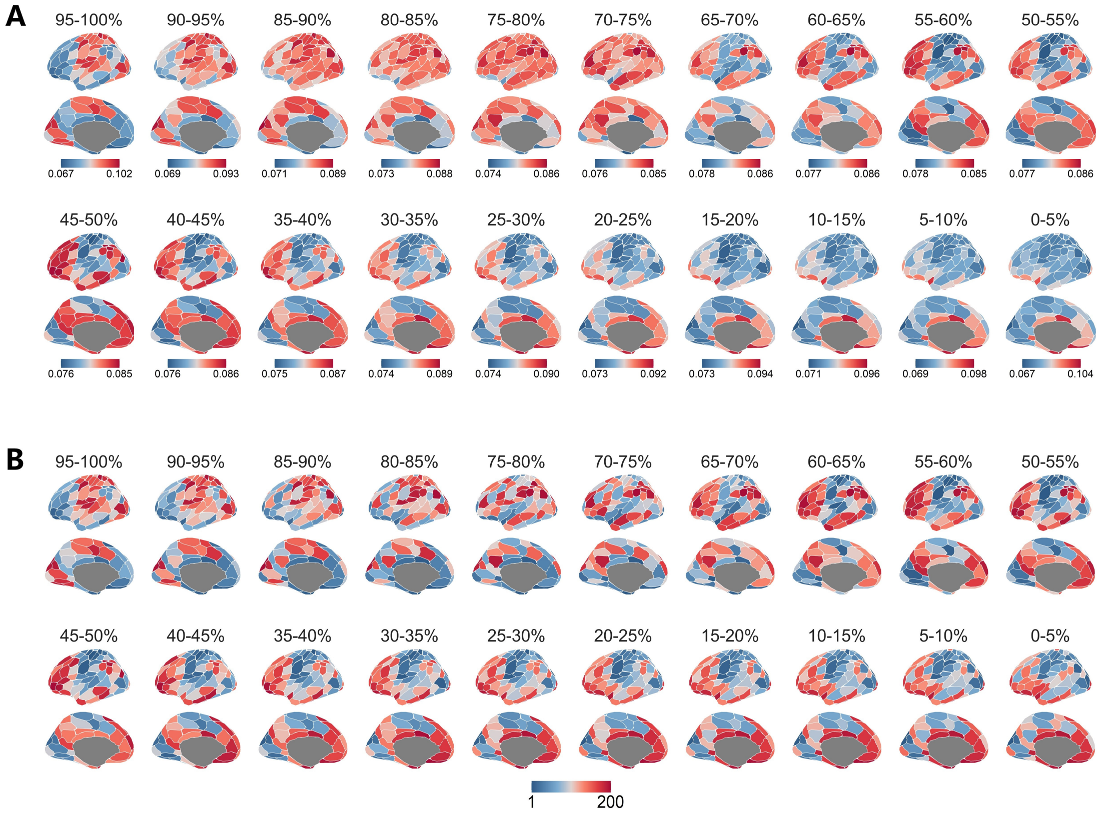

Fig. S2. Co-fluctuation score maps for 20 amplitude bins derived from the HCP 3T dataset following global signal regression. (**A**) Raw co-fluctuation scores for each bin. (**B**) To facilitate direct comparison of the hierarchy of co-fluctuation scores in the cerebral cortex, we plotted their ranks (from 1 to 200) for all bins.

bioRxiv preprint doi: https://doi.org/10.1101/2025.07.14.660681; this version posted July 16, 2025. The copyright holder for this preprint (which was not certified by peer review) is the author/funder, who has granted bioRxiv a license to display the preprint in perpetuity. It is made available under aCC-BY 4.0 International license.

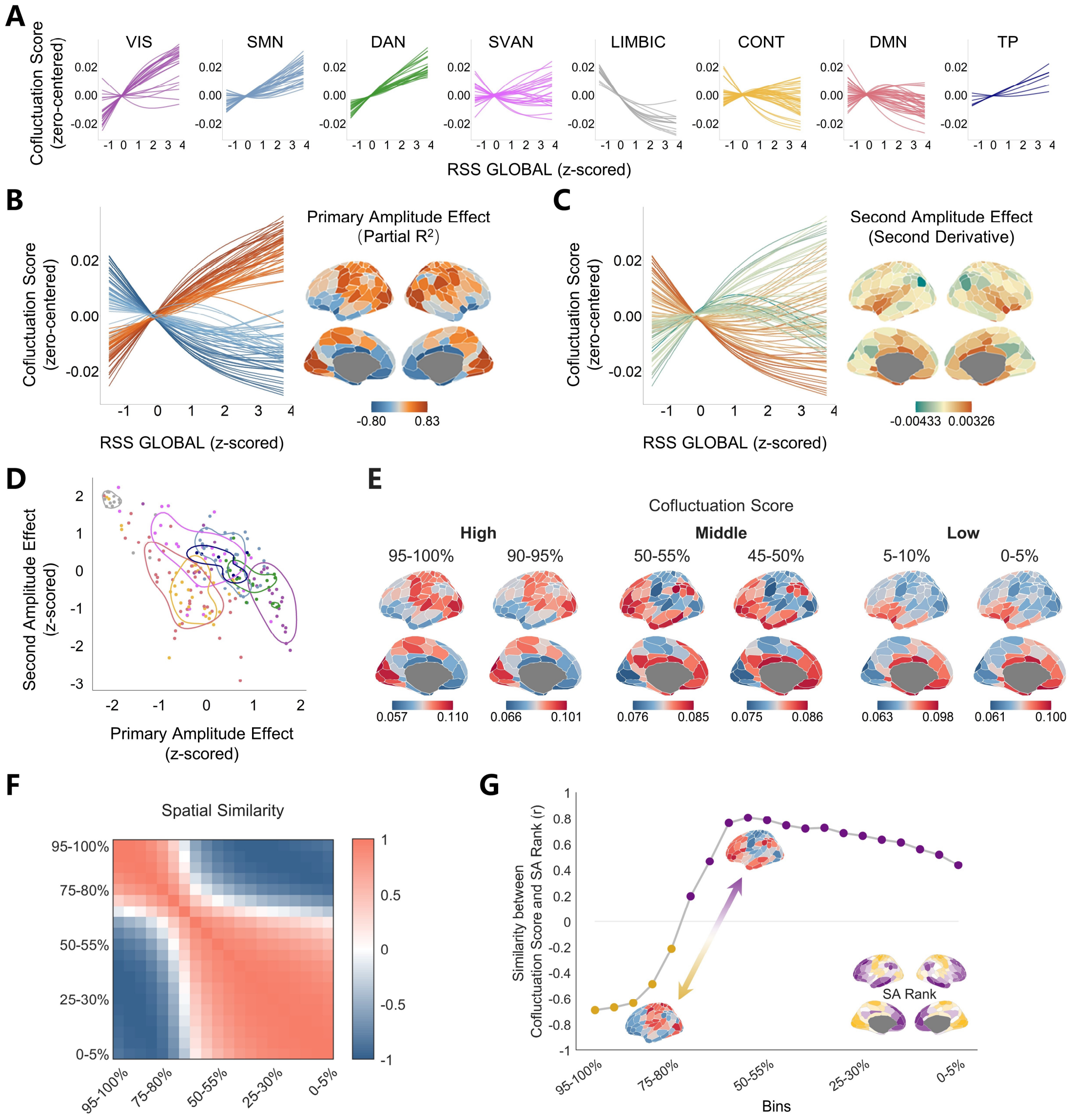

Fig. S3. Sensitivity analysis without global signal regression. The same analysis has been conducted using the same data but preprocessed without global signal regression. The findings could be replicated as in Fig. 2. (**A**) Co-fluctuation score trajectories were grouped for eight canonical functional networks. (**B**) The primary amplitude effect (partial R^2) summaries the overall changing trend of co-fluctuation scores with global amplitudes. (**C**) The second amplitude effect (averaged second-order derivatives) further characterizes the shape of trajectories. (**D**) The scatter plot of the primary and second amplitude effects. (**E**) The regional co-fluctuation score maps at the high, middle and low amplitude bins. (**F**) The similarity of spatial patterns of co-fluctuation scores across 20 bins (Pearson's correlation coefficient). (**G**) The correlation between co-fluctuation score maps and SA rank map at each bin (Spearman correlation coefficient).

bioRxiv preprint doi: https://doi.org/10.1101/2025.07.14.660681; this version posted July 16, 2025. The copyright holder for this preprint (which was not certified by peer review) is the author/funder, who has granted bioRxiv a license to display the preprint in perpetuity. It is made available under aCC-BY 4.0 International license.

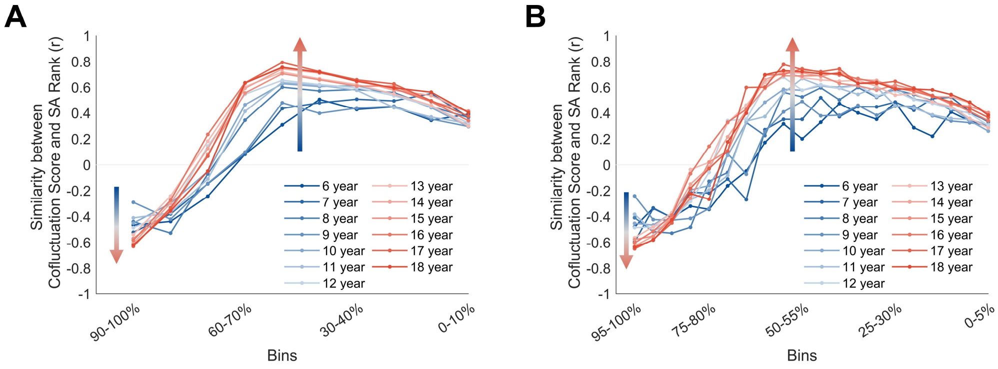

Fig. S4. Sensitivity analysis of the maturation of similarity between SA rank and co-fluctuation score. (**A**) We reorganize all frames into 10 bins (10% timepoints per bin) according their global RSSs. In this case, the number of timepoints is nearly twice that of 20 bins. (**B**) The raw similarity trajectories corresponding to Fig. 4C are provided.

bioRxiv preprint doi: https://doi.org/10.1101/2025.07.14.660681; this version posted July 16, 2025. The copyright holder for this preprint (which was not certified by peer review) is the author/funder, who has granted bioRxiv a license to display the preprint in perpetuity. It is made available under aCC-BY 4.0 International license.

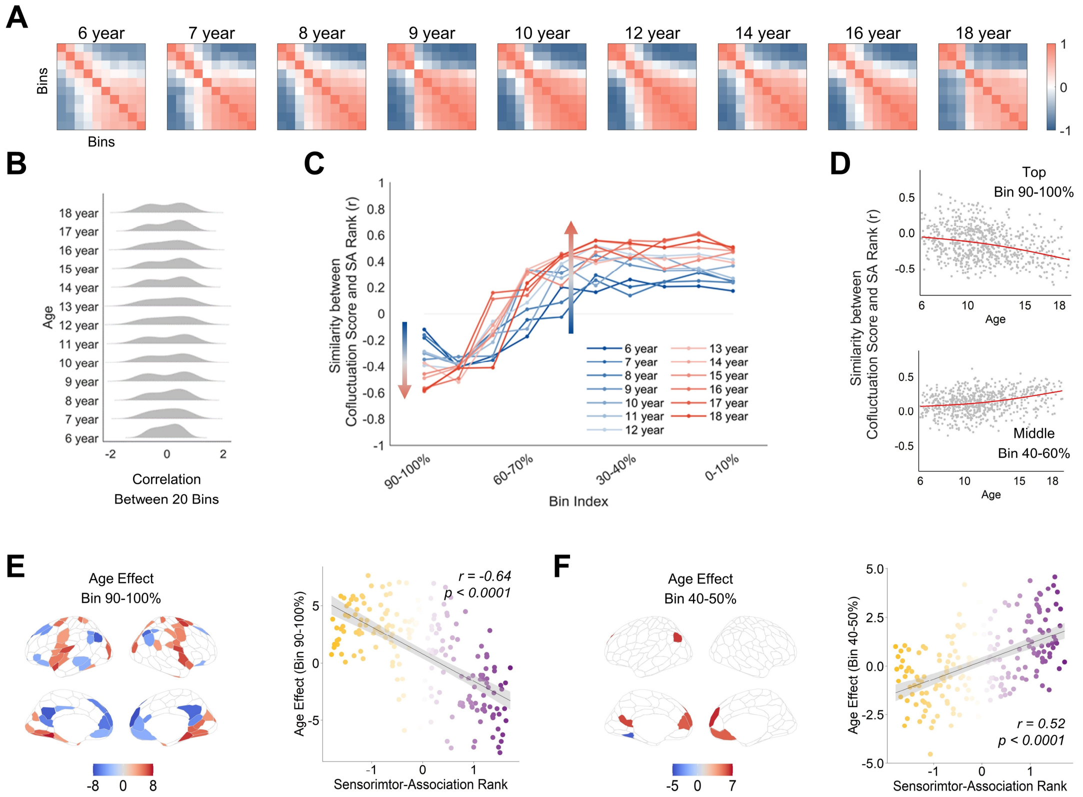

Fig. S5. The reproducibility of developmental findings using independent dataset (CCNP). Due to the short rs-fMRI scanning duration for the CCNP dataset, we set the number of bins to10 to ensure the stability of the results. The findings could be replicated as shown in Fig. 4. (**A**) Correlations of co-fluctuation score maps across 20 amplitude bins from 6 to 18 years old. (**B**) Distributions of correlations of co-fluctuation score maps across 20 bins, revealing increasing dissociation of two clusters from 6 to 18 years old. (**C**) Trajectories of amplitude-dependent alignment between co-fluctuation score maps and SA rank map approach adult-like pattern (grey line) from 6 to 18 years old. (**D**) The similarities between SA ranks and co-fluctuation score maps of individuals at high and intermediate amplitude bins. Each point represents an individual participant. (**E-F**) Left: Age-related effects on co-fluctuation scores at high- and intermediate-amplitude bins ($p < 0.005$, BHFDR corrected). Right: SA ranks predict age-related effects on co-fluctuation scores at high and intermediate amplitude bins.

bioRxiv preprint doi: https://doi.org/10.1101/2025.07.14.660681; this version posted July 16, 2025. The copyright holder for this preprint (which was not certified by peer review) is the author/funder, who has granted bioRxiv a license to display the preprint in perpetuity. It is made available under aCC-BY 4.0 International license.

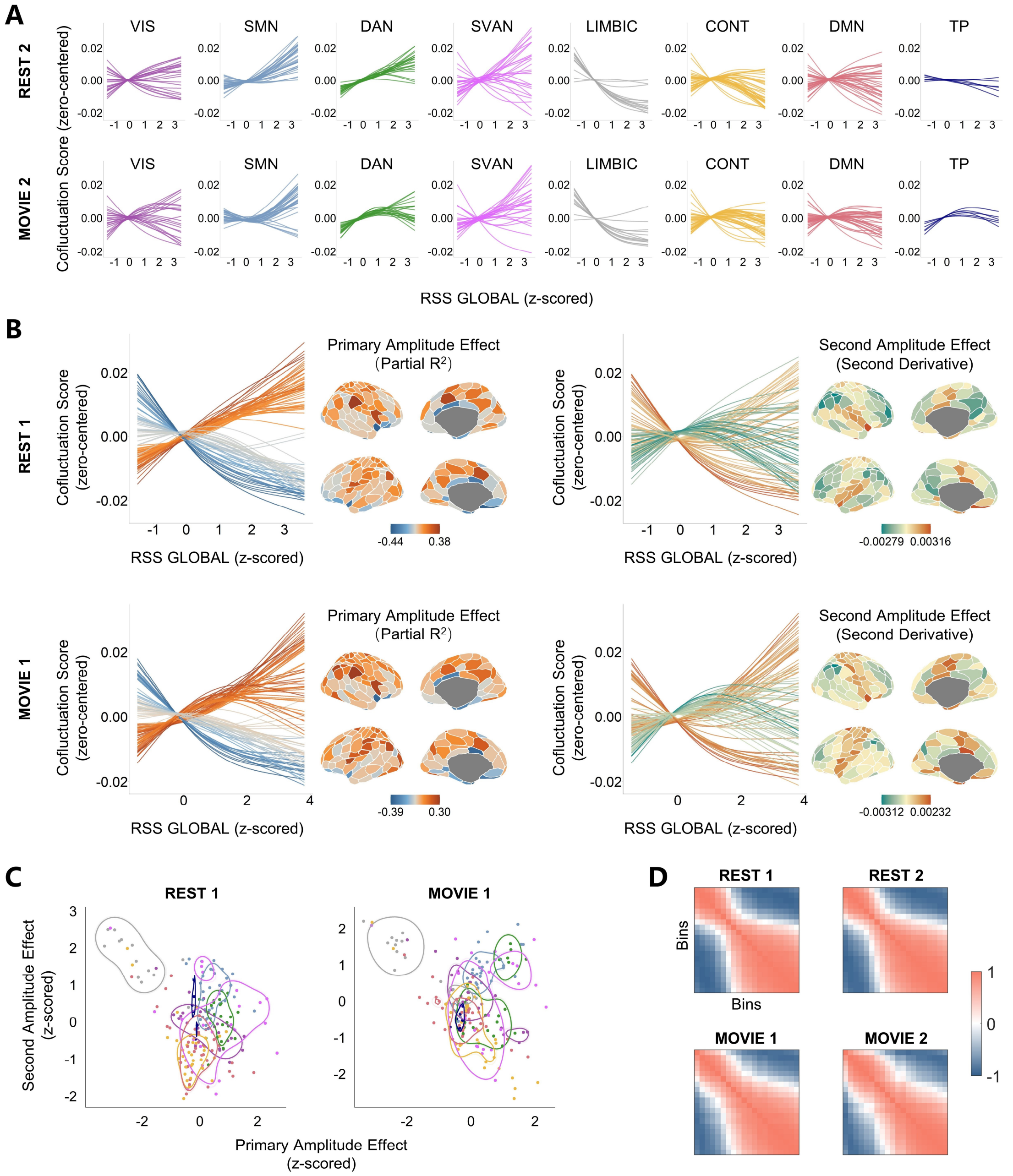

Fig. S6. Replication of the findings on adults using 7T fMRI data. (**A**) Regional trajectories of co-fluctuation scores of eight canonical functional networks during the second resting-state and movie-watching scans, derived from a 7T fMRI dataset. (**B-C**) The primary and second amplitude effects during the first resting-state and movie-watching sessions. (**D**) Similarities of co-fluctuation score maps across 20 amplitude bins under each of two resting-state and move-watching sessions.

bioRxiv preprint doi: https://doi.org/10.1101/2025.07.14.660681; this version posted July 16, 2025. The copyright holder for this preprint (which was not certified by peer review) is the author/funder, who has granted bioRxiv a license to display the preprint in perpetuity. It is made available under aCC-BY 4.0 International license.

Table S1. Age effects of regional co-fluctuation score at high-amplitude bin (90-100%).

Positive Age Effect			Negative Age Effect		
**ROIName**	**T**	**p**	**ROIName**	**T**	**p**
RH_SomMotB_S2_4	10.6	0	LH_LimbicB_OFC_1	-13.6	0
LH_DorsAttnA_ParOcc_1	10.5	0	RH_LimbicA_TempPole_3	-13.5	0
RH_DorsAttnB_PostC_1	9.6	0	LH_LimbicB_OFC_2	-12.7	0
RH_DorsAttnA_ParOcc_1	8.7	0	RH_LimbicB_OFC_2	-12.5	0
LH_VisCent_ExStr_1	8.7	0	RH_LimbicB_OFC_1	-12.4	0
LH_VisCent_ExStr_5	8.6	0	RH_DefaultA_PFCm_1	-11.9	0
LH_SomMotB_S2_3	8.5	0	LH_DefaultA_PFCd_1	-11.3	0
LH_VisPeri_ExStrInf_2	8.4	0	LH_LimbicA_TempPole_1	-11.2	0
LH_SomMotB_S2_2	8.2	4.44E-16	RH_ContB_PFCmp_1	-10.6	0
RH_VisCent_ExStr_1	8.1	8.88E-16	LH_DefaultB_PFCd_3	-10.5	0
LH_SomMotB_Cent_1	8.0	1.78E-15	LH_ContA_IPS_1	-10.3	0
RH_VisCent_ExStr_4	7.9	5.77E-15	RH_LimbicA_TempPole_2	-10.2	0
RH_SomMotB_Cent_1	7.8	6.22E-15	RH_ContB_IPL_2	-10.0	0
RH_VisCent_ExStr_5	7.8	7.99E-15	LH_DefaultA_PFCm_3	-9.9	0
RH_DorsAttnA_SPL_4	7.4	1.38E-13	LH_DefaultB_PFCd_4	-9.8	0
RH_VisPeri_ExStrInf_1	7.4	2.23E-13	RH_ContB_PFCld_2	-9.7	0
LH_VisCent_ExStr_4	7.4	2.33E-13	LH_DefaultB_PFCv_2	-9.7	0
LH_VisPeri_ExStrSup_2	7.1	1.28E-12	LH_ContA_PFCl_3	-9.7	0
LH_DorsAttnB_PostC_1	7.1	1.81E-12	LH_SalVentAttnB_PFCmp_1	-9.5	0
RH_VisPeri_ExStrSup_1	7.1	1.84E-12	RH_ContA_IPS_1	-9.5	0

bioRxiv preprint doi: https://doi.org/10.1101/2025.07.14.660681; this version posted July 16, 2025. The copyright holder for this preprint (which was not certified by peer review) is the author/funder, who has granted bioRxiv a license to display the preprint in perpetuity. It is made available under aCC-BY 4.0 International license.

Table S2. Age effects of regional co-fluctuation score at middle-amplitude bin (40-60%).

Positive Age Effect			Negative Age Effect		
**ROIName**	**T**	**p**	**ROIName**	**T**	**p**
RH_ContB_PFCmp_1	6.7	2.86E-11	LH_VisCent_ExStr_1	-5.2	2.72E-07
LH_ContA_PFCl_3	6.7	3.15E-11	LH_VisCent_ExStr_2	-5.0	7.84E-07
LH_DefaultB_PFCd_3	6.4	1.40E-10	LH_VisCent_Striate_1	-4.7	2.19E-06
LH_ContA_IPS_1	5.9	3.08E-09	LH_VisCent_ExStr_3	-4.4	1.12E-05
RH_ContB_PFCld_2	5.9	3.71E-09	LH_VisCent_ExStr_4	-4.4	1.37E-05
RH_ContB_IPL_2	5.7	1.21E-08	LH_VisCent_ExStr_5	-4.5	7.74E-06
RH_ContB_PFCld_1	5.6	1.95E-08	LH_VisPeri_ExStrInf_1	-3.2	0.0016
LH_DefaultB_PFCd_1	5.5	4.32E-08	LH_VisPeri_ExStrInf_2	-4.6	4.90E-06
LH_DefaultA_PFCd_1	5.5	4.47E-08	LH_SomMotA_6	-3.1	0.0018
RH_DefaultA_PFCm_3	5.4	7.37E-08	LH_SomMotB_S2_2	-5.0	5.01E-07
LH_ContB_PFCl_1	5.4	9.45E-08	LH_SomMotB_S2_3	-5.8	7.46E-09
LH_DefaultB_PFCd_4	5.3	1.44E-07	LH_SomMotB_Cent_1	-5.2	2.41E-07
RH_SomMotB_Aud_1	5.2	1.90E-07	LH_DorsAttnA_TempOcc_2	-4.0	5.44E-05
LH_ContB_IPL_1	5.1	3.52E-07	LH_DorsAttnA_ParOcc_1	-3.7	0.0002
RH_ContB_PFClv_1	4.9	8.27E-07	LH_DorsAttnA_SPL_3	-3.2	0.0013
RH_ContA_IPS_1	4.8	1.32E-06	LH_DorsAttnB_PostC_1	-6.4	2.32E-10
LH_ContA_PFCl_1	4.8	1.55E-06	LH_DorsAttnB_PostC_2	-5.3	1.19E-07
RH_DefaultA_PFCm_1	4.8	1.79E-06	LH_DorsAttnB_PostC_3	-3.0	0.0024
RH_SalVentAttnB_PFClv_1	4.5	6.50E-06	LH_LimbicA_TempPole_3	-5.4	5.63E-08
LH_ContA_IPS_2	4.5	6.98E-06	LH_LimbicA_TempPole_4	-5.6	2.72E-08

bioRxiv preprint doi: https://doi.org/10.1101/2025.07.14.660681; this version posted July 16, 2025. The copyright holder for this preprint (which was not certified by peer review) is the author/funder, who has granted bioRxiv a license to display the preprint in perpetuity. It is made available under aCC-BY 4.0 International license.

Table S3. Differences of regional cofluctuation score between movie and rest conditions at high-amplitude bin (90-100%).

**Movie > Rest**			**Movie < Rest**		
**ROIName**	**T**	**p**	**ROIName**	**T**	**p**
LH_VisCent_ExStr_3	22.1	4.49E-51	LH_VisPeri_ExStrSup_2	-19.6	1.88E-44
LH_ContC_pCun_1	18.8	3.43E-42	LH_VisPeri_ExStrInf_2	-19.5	6.23E-44
RH_ContB_PFCld_1	18.6	1.47E-41	RH_VisPeri_ExStrInf_1	-19.0	1.34E-42
LH_ContC_Cingp_1	18.3	1.08E-40	RH_VisPeri_ExStrSup_3	-16.6	6.43E-36
LH_VisCent_Striate_1	18.0	6.33E-40	RH_SalVentAttnA_Ins_2	-14.7	2.65E-30
RH_VisCent_Striate_1	17.7	4.21E-39	LH_DorsAttnB_FEF_1	-13.6	3.90E-27
RH_ContB_IPL_2	17.5	1.96E-38	RH_DorsAttnB_FEF_1	-13.0	1.56E-25
RH_ContC_Cingp_1	17.3	6.75E-38	RH_VisPeri_ExStrSup_2	-12.7	1.96E-24
RH_VisCent_ExStr_3	16.6	6.05E-36	RH_VisPeri_ExStrSup_1	-12.1	7.64E-23
RH_SalVentAttnB_PFCmp_1	15.3	2.89E-32	LH_SomMotB_Aud_3	-10.9	2.03E-19
RH_ContC_pCun_1	14.8	1.26E-30	LH_DorsAttnA_SPL_2	-10.9	2.11E-19
LH_SalVentAttnB_PFCmp_1	14.3	3.37E-29	LH_VisPeri_StriCal_1	-10.8	5.06E-19
RH_SalVentAttnB_Ins_1	13.7	1.93E-27	RH_DorsAttnB_PostC_3	-10.5	4.90E-18
LH_ContB_PFClv_2	13.3	2.61E-26	LH_VisPeri_ExStrSup_1	-10.3	1.43E-17
LH_ContA_IPS_1	12.1	7.73E-23	RH_DorsAttnA_SPL_3	-9.5	2.85E-15
RH_SalVentAttnB_IPL_1	12.1	8.27E-23	LH_SomMotB_Aud_2	-9.2	1.38E-14
RH_LimbicA_TempPole_4	12.1	1.02E-22	LH_DefaultA_pCunPCC_1	-9.2	1.91E-14
LH_VisCent_ExStr_1	11.4	7.52E-21	RH_LimbicB_OFC_3	-9.2	2.36E-14
LH_ContA_PFClv_1	11.0	1.15E-19	RH_ContB_Temp_1	-9.1	4.31E-14
LH_LimbicA_TempPole_2	9.8	3.38E-16	LH_DefaultA_IPL_1	-8.9	8.80E-14

bioRxiv preprint doi: https://doi.org/10.1101/2025.07.14.660681; this version posted July 16, 2025. The copyright holder for this preprint (which was not certified by peer review) is the author/funder, who has granted bioRxiv a license to display the preprint in perpetuity. It is made available under aCC-BY 4.0 International license.

Table S4. Differences of regional cofluctuation score between movie and rest conditions at middle-amplitude bin (40-60%).

Movie > Rest			Movie < Rest		
**ROIName**	**T**	**p**	**ROIName**	**T**	**p**
LH_VisPeri_ExStrInf_1	11.9	2.87E-22	LH_VisCent_Striate_1	-9.0	5.09E-14
RH_VisPeri_ExStrInf_1	10.1	4.61E-17	RH_ContB_PFCld_1	-8.8	1.91E-13
LH_VisPeri_ExStrInf_2	9.9	1.97E-16	LH_VisCent_ExStr_3	-8.6	9.83E-13
LH_VisPeri_ExStrSup_2	9.2	2.27E-14	RH_ContB_IPL_2	-7.6	2.91E-10
RH_DorsAttnB_FEF_1	8.7	5.39E-13	RH_VisCent_Striate_1	-7.3	1.42E-09
RH_VisPeri_ExStrSup_3	8.6	8.83E-13	RH_SalVentAttnB_PFCmp_1	-7.3	2.00E-09
LH_SomMotB_Aud_2	8.4	1.99E-12	RH_VisCent_ExStr_3	-7.0	1.25E-08
LH_DorsAttnA_SPL_2	8.3	3.62E-12	LH_ContA_IPS_1	-6.5	1.61E-07
RH_DorsAttnA_SPL_1	7.9	4.08E-11	RH_SalVentAttnB_PFCl_1	-6.5	1.90E-07
RH_VisPeri_ExStrSup_1	7.6	2.43E-10	RH_DefaultA_PFCm_1	-6.3	3.84E-07
RH_DefaultC_PHC_1	7.6	4.02E-10	LH_ContA_PFClv_1	-6.2	6.03E-07
LH_SomMotB_Aud_1	7.5	6.71E-10	LH_ContB_PFClv_2	-6.2	7.43E-07
RH_DorsAttnB_PostC_3	7.2	2.82E-09	RH_ContB_PFCmp_1	-6.2	9.43E-07
LH_SomMotB_Aud_3	7.1	5.55E-09	RH_SalVentAttnB_IPL_1	-6.1	1.15E-06
RH_SomMotB_Aud_2	7.1	6.19E-09	RH_ContC_Cingp_1	-6.1	1.25E-06
LH_DorsAttnA_ParOcc_1	7.0	9.57E-09	LH_ContC_pCun_1	-6.1	1.27E-06
RH_VisPeri_ExStrSup_2	6.8	2.37E-08	LH_SalVentAttnB_PFCmp_1	-6.0	1.99E-06
LH_DorsAttnB_FEF_1	6.8	2.52E-08	RH_ContC_pCun_1	-5.6	1.50E-05
RH_DorsAttnA_SPL_4	6.7	5.73E-08	RH_DefaultB_PFCv_1	-5.2	9.37E-05
RH_SomMotB_Aud_1	6.5	1.25E-07	LH_SalVentAttnB_PFCl_1	-5.2	0.0001
